# ColorViz, a New and Rapid Tool for Assessing Collateral Circulation during Stroke

**DOI:** 10.3390/brainsci10110882

**Published:** 2020-11-20

**Authors:** Tommaso Verdolotti, Fabio Pilato, Simone Cottonaro, Edoardo Monelli, Carolina Giordano, Pamela Guadalupi, Massimo Benenati, Antonia Ramaglia, Alessandro Maria Costantini, Andrea Alexandre, Riccardo Di Iorio, Cesare Colosimo

**Affiliations:** 1 UOC Radiologia e Neuroradiologia, Fondazione Policlinico Universitario A. Gemelli IRCCS, 00168 Rome, Italy; alessandromaria.costantini@policlinicogemelli.it (A.M.C.); andrea.alexandre@policlinicogemelli.it (A.A.); cesare.colosimo@policlinicogemelli.it (C.C.); 2 Neurology, Neurophysiology and Neurobiology Unit, Department of Medicine, Università Campus bio-medico di Roma, 00128 Rome, Italy; fabio.pilato@policlinicogemelli.it; 3 Dipartimento di Diagnostica per Immagini, Università Cattolica del Sacro Cuore, Istituto di Radiologia, 00168 Rome, Italy; cottonaro.simone90@gmail.com (S.C.); monedoardo@gmail.com (E.M.); carolinagiordano91@gmail.com (C.G.); pamelaguadalupi@gmail.com (P.G.); 4 Dipartimento di Diagnostica per Immagini, Radioterapia, Oncologia ed Ematologia, Fondazione Policlinico Universitario A. Gemelli IRCCS, 00168 Rome, Italy; dr.massimo.benenati@gmail.com (M.B.); ramaglia88@gmail.com (A.R.); 5 Neurology Unit, Fondazione Policlinico Universitario A. Gemelli IRCCS, 00168 Rome, Italy; riccardo.diiorio@policlinicogemelli.it

**Keywords:** acute ischemic stroke, multiphase CT angiography, leptomeningeal collaterals, Color Map, Collateral grading scales

## Abstract

Prognosis of patients with acute ischemic stroke is strictly related to the patency and prominence of the collateral leptomeningeal pathways distal to the arterial occlusion. The gold standard for assessment of collateral circulation is conventional angiography, but it is invasive and used in selected cases. To date, the most reliable technique is multiphase CTA; currently, the available classifications of collateral circles are often complex, time-consuming, and require a trained observer. The purpose of our work is to establish the effectiveness of a new semi-automatic post-processing software (ColorViz FastStroke, GE Healthcare, Milwaukee, Wisconsin) in evaluation of collateral circulation compared to the six-point classifications of multiphase CTA already validated in literature. We selected 86 patients with anterior ischemic stroke symptoms who underwent multiphasic CTA in our emergency department. Two radiologists separately evaluated the collateral leptomeningeal vessels, analyzing respectively, the multiphase CTA (using the six-point scale and its trichotomized form) and ColorViz (using a three-point scale). Then the results were matched. We found a good correlation between the two different analyses; the main advantage of ColorViz is that, while maintaining fast diagnostic times, it allows a simpler and more immediate evaluation of collateral circulation, especially for less experienced radiologists.

## 1. Introduction

Acute ischemic stroke (AIS) is a time-dependent medical emergency whose evaluation must be fast and careful at the same time in order to select which patients can be eligible for specific life-saving treatments [1]. It has been calculated that every 60 min delay, starting 3.5 h after symptom onset, reduces by 20% the chance of achieving a recovery of functional independence [2].

The three compartments model best describes the need to act as fast as possible in case of large vessel occlusion (LVO). The central area (called “core”) is the one that cannot be saved under any circumstances; the intermediate zone (penumbra) is the one potentially recoverable through a prompt intervention by intravenous thrombectomy (IVT) and mechanical thrombectomy (MT); finally, the outermost zone (“oligoemia” zone) is the one in which recovery should take place even in the absence of any treatments [2]. It is logical to understand that a faster image acquisition will result in a better outcome for the patient.

CT scan is the modality of choice to achieve these goals, as it allows a rapid assessment of the patient with AIS and is widely available in territory [3]. Non-enhanced CT (NeCT) allows exclusion of intraparenchymal hemorrhages (contraindication for IVT) and stroke mimics; it is also able to recognize the signs of ischemia and, in some cases, localize the clot [2,3,4]. Multiphase CT angiography (mCTA) allows better localization of arterial occlusion and provides a spatial and temporal evaluation of the patency and status of the collateral circulation [1,5,6].

There are many studies that show how the presence of good leptomeningeal collaterals allows maintenance of an adequate blood flow towards the ischemic penumbra (to make a subsequent attempt at reperfusion more effective) [4,7], which also increases the probability of a better outcome for the patient [6,7,8,9].

In diagnostic practice, it is also important to quickly establish the status of the collateral circulation downstream of occlusion to select as quickly as possible patients eligible for thrombectomy [1,4,10].

In literature, there exists a lot of scoring scales for evaluating collateral circulation in mCTA, but many of them are complex and none are standardized for clinical practice [1,2,11].

The color-coded map (ColorViz) is a rapid, clear, and more immediate post-processing tool that permits maintenance of the temporal resolution of mCTA, summing in a single image the three different cerebral vascular phases using a time variant color map [10]. Recently, Ospel et al. have already shown potential applications of ColorViz maps when assessing patients with stroke [10], but a systematic analysis of inter-reader agreement was not performed.

The aim of our study is to compare the real diagnostic reproducibility between scores obtained through ColorViz compared to those obtained through mCTA in judging the state of collateral circulation during anterior LVO.

## 2. Material and Methods

A retrospective observational study was designed. Starting from 1 January 2018, we interrogated our institutional database by inserting the keywords “anterior circulation ischemic stroke”. We selected only patients who underwent CT examination protocol for stroke (including NeCT and mCTA) in our hospital. We excluded two patients because, during the evaluation of the colorimetric map, the color of the healthy hemisphere was overall altered (green or blue), without an appreciable difference in terms of delayed circulation compared to the contralateral affected side. Finally, 86 patients (37 M, 49 F) were enrolled.

A portion of the selected patients underwent CT protocol with additional perfusion CT scan (PCT). For each patient that was included in our protocol, the following clinical information was collected: NIHSS at the onset, symptoms at the onset, and supposed time of stroke onset.

Forty-six (54%) patients were subsequently treated with endovascular treatment (thrombectomy), thrombolysis, or both. Ethics approval: The local institutional review board approved the study (study protocol 6410/20, ID 3004).

### 2.1. Image Acquisition and CT Protocol

All patients underwent multimodal CT imaging, including NeCT and mCTA, performed as described in literature [1,4,5].

Briefly, CT imaging was performed using a 64-multislice CT (GE MEDICAL SYSTEM Optima CT660 645, GE Healthcare, Milwaukee, Wisconsin).

Acquisition parameters for NeCT were 120 kv and 44mAs, and acquisition duration was 9 s.

Acquisition parameters for mCTA were 100 kV and 4 mAs, and acquisition duration was 21 s; after administration of 80 mL of contrast media at 4 mL/s flow, the acquisition was composed of three subsequent phases (separated by an interval of 8 s): the first phase (acquired in the arterial phase) extends from the aortic arch to the vertex, and the next two phases (acquired in the early and late venous phases) from the occipital foramen to the vertex.

In selected cases in which it was indicated, CTP was performed with an 8 cm *z*-axis coverage after administration of rapid infusion of 40 mL before 70 mL with a flow of 4 mL/s.

### 2.2. Software Functioning

The color-coded mCTA summation maps were obtained on a workstation using FastStroke software (GE Healthcare, Milwaukee, WI, USA) that elaborates the full set of images included in a CT stroke protocol (NeCT, mCTA) into one single color-coded map called ColorViz.

The vessels displayed on the map appeared differently colored based on the arrival time of the contrast medium and on a per-person adaptive threshold technique [10]. In detail, the colors assigned to vessels can be red (to those that enhance maximally during the arterial phase), green (to those that enhance in the early venous phase), and blue (to those that enhance in the late venous phase). Post-processing is easy and fully automated, and it can be shown in about 10–15 s.

### 2.3. Image Interpretation

Two neuroradiologists (S.C. and E.M.) separately evaluated the site of the anterior LVO and the status of collateral leptomeningeal vessels, analyzing respectively the mCTA and color-coded summation maps generated for each patient, and rating the status of collateral leptomeningeal vessels.

In the evaluation of mCTA, we assessed the extension and delay in the pial arterial filling in the affected side compared to the healthy contralateral one using a six-point score (then simplified in its trichotomized form) [5]. For the analysis of ColorViz, we established a three-point qualitative scale, dividing collateral status into poor, intermediate, or good, which recalled the trichotomized form of the six-point scale of mCTA (Table 1).

In this case, we considered the prevalence and predominant color of the collateral circulation in the affected side with respect to the contralateral one.

**Figure 1:**
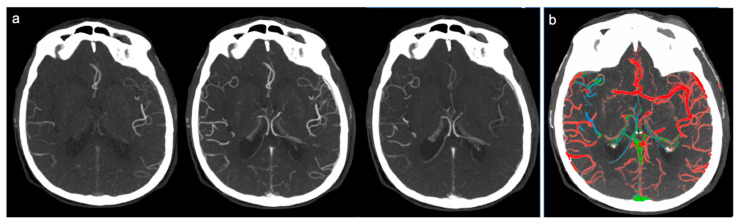
Example of good collateral circulation at ColorViz. (**a**) mCTA shows a right-sided M1 segment occlusion with good pial artery filling (1 phase delay); (**b**) ColorViz map shows predominant red vessels in the affected territory downstream from the occlusion (score of 3).

**Figure 2:**
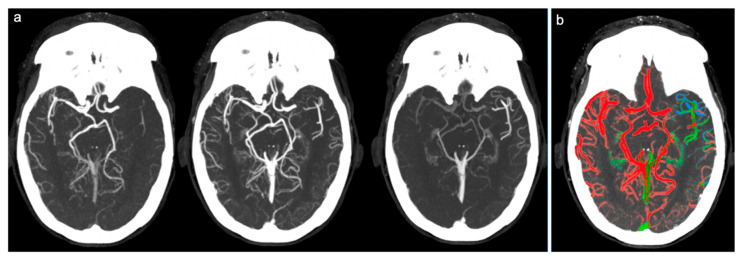
Example of intermediate collateral circulation at ColorViz. (**a**) mCTA shows a left-sided M1 segment occlusion with intermediate pial artery filling; (**b**) ColorViz map shows predominant green vessels in the affected territory downstream from the occlusion (score of 2).

**Figure 3:**
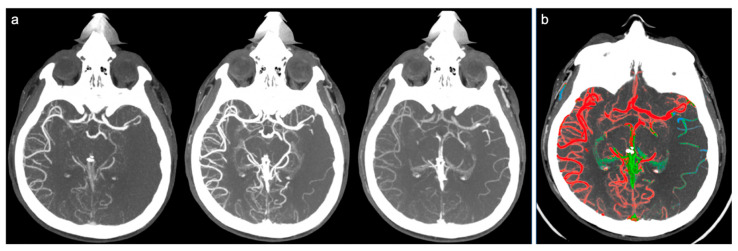
Example of poor collateral circulation at ColorViz. (**a**) mCTA shows a left-sided M1 segment occlusion with poor pial artery filling; (**b**) ColorViz map shows reduced extension of collateral circles vessels in the affected territory downstream from the occlusion (score of 1).

Therefore, the collateral circulation was defined as “good” and a score of 3 was assigned in cases where a good or slightly reduced representation of the collateral circles was found on the affected side and the predominant color was red (Figure 1). Score 2 (“intermediate”) was assigned in cases where a similar or reduced extension of collateral circulation was found compared to the healthy side, but the predominant color was green (slightly slowed circulation) (Figure 2). Finally, a score of 1 (“poor”) was given in cases where the most prevalent color was blue (significantly slowed circulation compared to the contralateral) or in cases in which the extension of collateral circles was significantly reduced (even in the presence of few green or red vessels) (Figure 3).

## 3. Statistical Analysis

All statistics were presented as mean and SD for continuous variables with a normal distribution, median and interquartile range for continuous variables with a non-normal distribution, and frequency and percentages for categorical variables. Univariate analysis was performed to assess differences between groups. Continuous variables with normal distribution were compared with the Student’s t-test, while the Wilcoxon rank sum test was used for continuous variables with a non-normal distribution. Categorical variables were compared by means of X^2^ test or Fisher’s exact test as appropriate. To assess the accordance between techniques and operators, a Pearson’s correlation test was used.

Analysis was performed with Statistical Package for Social Sciences (IBM SPSS Version 25.0.IBM Corp, Armonk, NY, USA.).

## 4. Results

Patient characteristics are reported in Table 2.

**Figure 4:**
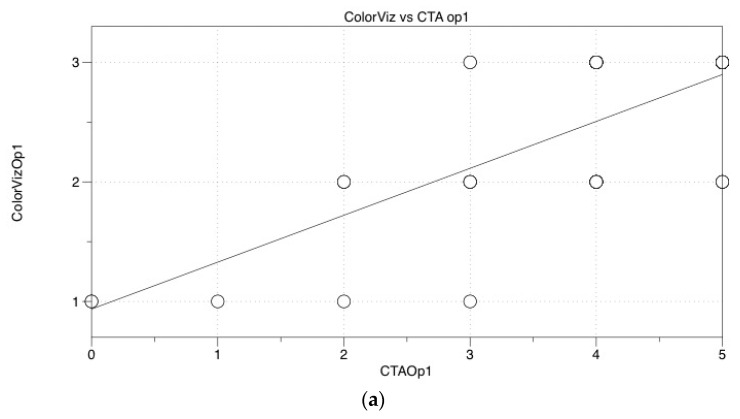

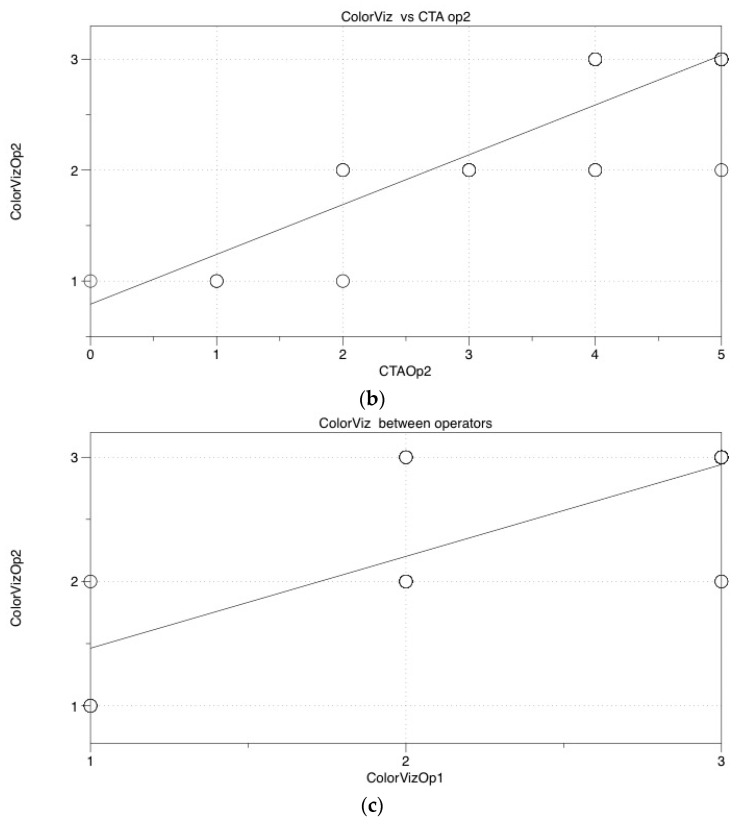
Statistical Graphs. (**a**,**b**), Graphs showing good accordance for both operators when comparing ColorViz to mCTA (*r* = 0.735, *p* < 0.001 and *r* = 0.839, *p* < 0.001, respectively); (**c**) Graph showing good accordance between operators when comparing ColorViz scores (*r* = 0.890, *p* < 0.001).

Evaluation by ColorViz showed good accordance for both operators (*r* = 0.735, *p* < 0.001 and *r* = 0.839, *p* < 0.001, respectively) compared to CTA (Figure 4a,b).

Moreover, we found a good accordance among operators when evaluating CTA (*r* = 0.962, *p* < 0.001) and Colorviz images (*r* = 0.890, *p* < 0.001) (Figure 4c).

## 5. Discussion

Numerous studies have shown how the presence of good collateral circulation in the ischemic area results in a smaller core volume, wider therapeutic window for the patient, and a better functional outcome at discharge [1,6,7,12,13,14]

The gold standard in the evaluation of collateral circles is digital subtraction angiography (DSA), which appears to be an invasive method, involves a considerable waste of economic and human resources, and is not so much diffused in the territory [1,5,11,15]. For these reasons, in the last 10 years, the mCTA has increasingly established itself in daily practice as a reference method. In the emergency setting, the main advantages are to get a good definition of the site of occlusion quickly and, at the same time, an evaluation of the state of collateral circulation downstream from the clot [2,5,6].

An adequate diagnostic protocol must include a non-contrast scan of the brain, followed by three consecutive scans, the first from the aortic arch to the vertex (arterial phase), and the second and third including only the head (in the early venous and late venous phase, respectively) approximately 5–10 sec from each other. This modality of acquisition is useful to obtain a time-resolved valuation of collateral circles and to demonstrate a delayed filling in the ischemic side [1,2,7].

Nowadays, one of the emerging main limitations of the mCTA in the ongoing assessment of AIS is the lack of standardized tools for the evaluation of the score of leptomeningeal circles downstream from the occlusion [1,2].

There are many classifications of collateral circle status published in the literature, but many of them include six-point scales, which have results often so complex that they require advanced neuroradiological skills. In addition, radiologists need to evaluate three different images simultaneously in mCTA, which implicates some degree of expertise, thus leading to a considerable waste of time during the diagnostic process [1].

For these reasons, we tried to compare mCTA with a new post-processing tool that allows to create, quickly and in a more immediate and clear visual impact, an image, called ColorViz, that merges the three phases of the CTA into a single phase [10]. The latter aspect has appeared to be of considerable interest, since it permits maintenance from the temporal resolution offered by mCTA using a time-variant color map and avoids the need to compare three different phases at the same time, thus making diagnostic evaluation faster and results more understandable for stroke clinicians involved both in stroke diagnosis and treatment. On the other hand, this time-saving procedure may be helpful in acute stroke evaluation because it allows a faster activation of required treatments, for example, mechanical thrombectomy.

The rationale of our work is to assess the reproducibility of this method compared to the one currently in use, evaluating its advantages and possible disadvantages. We also tried to use an easier score based on a three-point scale to make assessment faster for radiologists.

From our experience, it emerged that the ColorViz was statistically comparable with respect to the mCTA (performed in urgency) with a good inter-reader agreement. We also found a great linear correlation between radiologists in evaluating color-coded maps.

In this perspective, the new territorial organization for stroke care and the nascent need to send images between HUB and SPOKE makes ColorViz particularly accessible even for less experienced radiologists.

A limitation of the study was that both the mCTA and the ColorViz analyses were not conducted in emergency conditions, which could have influenced both the decisions of the reviewing radiologist and the diagnostic times. Despite this premise, both the post-processing times (about 10–15 s) and especially the diagnostic times appeared overall faster in ColorViz than in mCTA.

In the near future, further studies carried out in vivo (under stress conditions of a real emergency) could confirm the real reproducibility and rapidity of the method.

Another study limit was the number of recruited patients; these results have to be replicated in a larger population of stroke patients with anterior circulation.

In a historical moment in which the definition of the status of ongoing AIS collateral circles is increasingly important [1,15], in the absence of scoring scales validated in clinical practice, it is necessary to direct efforts towards the use of methods that make the diagnostic evaluation more immediate and reproducible for radiologists.

## 6. Conclusions

The ColorViz has proved to be an effective tool in the evaluation of collateral circulation, proving to be comparable in terms of diagnostic evaluation to the mCTA. The possibility of using a simpler and more immediate scoring scale for ColorViz than those present in the literature for the mCTA made the diagnostic evaluation faster and easier for the radiologist.

## Figures and Tables

**Table 1:** ColorViz scores of collateral status.

ColorViz—Description of Findings	ColorViz Three-Point Scale
Good or slightly reduced representation of collateral circulation and prevalence of red vessels downstream from the occlusion.	3 GOOD
Same or reduced extension of collateral circulation, but with prevalence of green vessels downstream from the occlusion.	2 INTERMEDIATE
Prevalence of blue vessels or significative reduction of collateral circulation downstream from the occlusion.	1 POOR

**Table 2:** Patients characteristics.

	ColorViz 3	ColorViz 2	ColorViz 1
**Patients** (*n*)	49 (31 F; 18 M)	28 (15 F; 13 M)	9 (2 F; 7 M)
**Age** (y.o.) *	77 (± 10)	77 (± 10)	76 (± 14)
**NeCT Aspect** **	9 (4–10)	7 (3–10)	6 (4–9)
**NIHSS onset** **	11 (1–25; tot 46 ^)	15 (6–25; tot 21 ^)	19 (8–23; tot 5 ^)
**Site of occlusion**:	9 (18.4%)	7 (25%)	2 (22.2%)
ICA	11 (22.4%)	17 (60.7%)	6 (66.7%)
M1	17 (34.7%)	4 (14.3%)	1 (11.1%)
M2	10 (20.4%)	0	0
Distal/not detected			
Emodinamic stroke	2 (4.1%)	0	0

* Age values are indicated as average; standard deviation is shown in brackets. ** NeCT and NIHSS values are indicated as median; min and max values are shown in brackets. ^ NIHSS median values were derived from 72 of the 86 total patients.
